# Influence of Musical Training on Understanding Voiced and Whispered Speech in Noise

**DOI:** 10.1371/journal.pone.0086980

**Published:** 2014-01-28

**Authors:** Dorea R. Ruggles, Richard L. Freyman, Andrew J. Oxenham

**Affiliations:** 1 Department of Psychology, University of Minnesota, Minneapolis, Minnesota, United States of America; 2 Department of Communication Disorders, University of Massachusetts, Amherst, Amherst, Massachusetts, United States of America; 3 Department of Psychology, University of Minnesota, Minneapolis, Minnesota, United States of America; University of Salamanca- Institute for Neuroscience of Castille and Leon and Medical School, Spain

## Abstract

This study tested the hypothesis that the previously reported advantage of musicians over non-musicians in understanding speech in noise arises from more efficient or robust coding of periodic voiced speech, particularly in fluctuating backgrounds. Speech intelligibility was measured in listeners with extensive musical training, and in those with very little musical training or experience, using normal (voiced) or whispered (unvoiced) grammatically correct nonsense sentences in noise that was spectrally shaped to match the long-term spectrum of the speech, and was either continuous or gated with a 16-Hz square wave. Performance was also measured in clinical speech-in-noise tests and in pitch discrimination. Musicians exhibited enhanced pitch discrimination, as expected. However, no systematic or statistically significant advantage for musicians over non-musicians was found in understanding either voiced or whispered sentences in either continuous or gated noise. Musicians also showed no statistically significant advantage in the clinical speech-in-noise tests. Overall, the results provide no evidence for a significant difference between young adult musicians and non-musicians in their ability to understand speech in noise.

## Introduction

Individuals with early and extensive musical training have been found to exhibit certain enhanced behavioral auditory abilities. One of the most robust auditory advantages demonstrated by musicians over non-musicians is in pitch discrimination [1]–[3]. Given the strong reliance of Western musical traditions on pitch, this advantage is perhaps not surprising, although non-musicians tend to reach the same performance levels as professional musicians after about 6–8 hours of training in pitch discrimination [3]. The acoustic correlate of pitch is stimulus periodicity, and several studies have identified enhanced responses to periodic stimuli in musicians with a physiological measure of sub-cortical phase locking known as the frequency following response (FFR), as well as with early cortical responses [4]–[6]. A relationship between FFR and pitch discrimination has been suggested by the finding of enhancement in both measures following a period of perceptual training in pitch discrimination [7].

Music is not alone in incorporating periodic sounds. The periodicity in voiced speech sounds, such as vowels, elicits a pitch sensation that carries important information about prosody and segmentation in non-tone languages, such as English, and carries lexical information in tone languages, such as Chinese. A link between speech perception and subcortical periodicity coding has been suggested by the finding that native speakers of Mandarin Chinese tend to have a stronger and more robust FFR to tone words than do native speakers of American English [8]. Even in non-tone languages, the periodicity information in voiced speech can affect the intelligibility of speech. For instance, voiced speech is more intelligible than whispered speech when presented in noise [9], and altering the natural periodicity (or fundamental-frequency, F0) contour of voiced speech can impair its intelligibility in noise [10], [11].

The connections between periodicity coding and speech intelligibility on one hand, and between musical training and pitch perception on the other, suggests a possible association between musical training and speech understanding: musical training may lead to enhanced neural coding of periodicity, which in turn may lead to enhanced perception of speech in noise [6], [12], [13]. Consistent with this hypothesis, Parbery-Clark et al. [14], [15] demonstrated a small but significant performance advantage for young adult musicians over non-musicians in two clinical tests of speech understanding in noise, and later related the musicians’ perceptual advantage to their larger and more robust neural responses to periodicity, as measured by the FFR [16].

A further benefit of periodicity coding for speech understanding has been posited for cases in which the background noise is fluctuating. It has been suggested that the segregation of speech from a fluctuating masker is aided by the periodicity in the voiced speech [17]. According to this hypothesis, the “masking release,” or improvement in performance when a steady-state noise is replaced by a fluctuating noise with the same overall sound level, is dependent in part on the neural coding of the periodic temporal fine structure (TFS) within speech [17]–[20]. Although the link between TFS coding and masking release has been questioned by recent studies [9], [21], [22], it remains true that differences in performance between conditions or between subject groups are often accentuated in a fluctuating noise [23], [24], so that any benefit of periodicity coding due to musical training should be further enhanced in a fluctuating masker.

The aim of this study was to test the hypothesis that musical training leads to improved speech understanding in noise because of enhanced periodicity coding. If the hypothesis is correct, then the speech-in-noise benefit found previously for musicians using normal (voiced) speech should not be found using whispered speech, due to its lack of periodicity. We tested the intelligibility of normal (voiced) and whispered speech in steady-state noise, as well as in amplitude-modulated, or gated, noise. We reasoned that any differences between musicians and non-musicians in speech intelligibility might be amplified in modulated noise, as found in earlier studies comparing normal-hearing listeners with hearing-impaired listeners [25], [26] or cochlear-implant users [27]. Experiment 1 investigates the effects of musical training on the intelligibility of voiced and whispered speech in stationary and modulated noise, and Experiment 2 further tests group differences using clinical speech-in-noise tests and a measure of pitch discrimination.

## Experiment 1

### Methods

#### Ethics statement

All methods were approved by the University of Minnesota Institutional Review Board, and all participants provided written informed consent.

#### Subjects

Thirty-three young adult listeners (18 female, age range 18–31 years, mean 21.2 years) were recruited from the University of Minnesota student population. Seventeen of these (10 female, mean age 20.7 years) had less than 2 years of formal musical training and did not report currently playing a musical instrument or taking active part in any musical activity, and so were categorized as non-musicians (NM). Sixteen of the listeners (8 female, mean age 21.8 years) had at least 10 years of formal musical training (10–22 years), beginning before the age of 10, and had consistently played a musical instrument since then, including currently playing at least 5 hours a week. These subjects were categorized as musicians (M; see Table 1 for details). Nearly all individuals categorized as musicians were enrolled in the University of Minnesota’s School of Music at the time of their participation.

**Table 1:** List of musicians, age at onset of musical training, and primary instrument.

Subject	Age of onset (yr)	Primary instrument
M1	7	Bass trombone
M2	7	Viola
M3 ^1^	5	Violin
M4	8	Bassoon
M5	5	Violin
M6	10	Cello
M7 ^1^	6	Voice
M8 ^1^	4	Voice
M9	9	Guitar
M10	7	Double bass
M11	9	Viola
M12	9	Flute
M13	7	Cello
M14	5	Trombone
M15	6	Violin
M16	6	Percussion
M17 ^2^	8	Clarinet

Musicians who only participated in Experiment 1 are indicated by a superscript 1, and those who only participated in Experiment 2 are indicated by a superscript 2. All others participated in both experiments.

All listeners had normal hearing, defined as audiometric thresholds of less than 20 dB HL at octave frequencies between 250 and 8000 Hz. A mixed-model repeated-measures ANOVA indicated no main effect of training group on auditory thresholds [F(1,207) = 1.3, p = 0.25] and no main effects of, or interactions with, frequency or ear (left vs right; p>0.06 in all cases).

All listeners completed the Vocabulary and Matrix Reasoning subtests of the Wechsler Abbreviated Scale of Intelligence – Second Edition (WASI-II; [28]). Scores from these two tests were combined to determine full scale IQ scores, which differed somewhat between groups [musician mean: 126.3; non-musician mean: 115.3; t(31) = 2.91, p = 0.007, d = 1.0].

#### Stimuli

The intelligibility of speech in background noise was measured for normal (voiced) and whispered (unvoiced) speech in the presence of stationary (continuous) and modulated (gated) noise at three different signal-to-noise ratios (SNRs). Nonsense sentences, developed by Helfer [29] and recorded by Freyman et al. [9], were used. Whispered speech was used, as opposed to vocoded speech, because it is naturally produced (avoiding vocoding artifacts) and does not confound lack of voicing with poorer spectral resolution [9].

In addition to presenting the voiced and whispered sentences recorded by Freyman et al. [9], a third speech condition was generated, using the whispered speech, but adjusting its spectro-temporal envelope distribution to more closely match that of the voiced speech. In contrast to the broadband adjustments made by Freyman et al. [9], the speech materials were first filtered into 1/3-octave subbands with center frequencies between 50 and 8000 Hz. The whispered and voiced sentences were concatenated into two separate arrays before filtering, and then each array was divided into 50-ms Hanning-windowed segments with 50% overlap. The rms amplitudes of the segments within the two arrays were computed and ranked within each frequency subband. Then the rms amplitudes of the whispered-speech segments were scaled to match the rms amplitude of the voiced-speech segment with the same rank in the same subband. Following the rescaling of the whispered-speech segments, they were recombined within subbands, and then the subbands were summed to produce adjusted whispered-speech sentences with approximately the same long-term spectrum and temporal envelope distribution as the original voiced speech. The main differences are that the voiced speech (and hence the adjusted whispered speech) has a greater spectral tilt than the whispered speech, and has a wider range of amplitude fluctuations. Both these differences reflect the higher energy in the low-frequency voiced speech. Despite these changes, the final adjusted whispered-speech sentences sounded very similar to the original whispered-speech sentences, with what could be described as a slight coloration. [Supplementary materials include example sound files.].

The long-term amplitude spectrum of the noise was matched to that of the speech type presented in a given block. The noise was either continuous or square-wave gated with a 50%, 16-Hz duty cycle. The speech was presented at 65 dB SPL, and the noise level was adjusted to achieve test SNRs of −6 dB, −3 dB, and 0 dB. Noise levels were set after the gating was applied, so that the noise level during the “on” periods of each square-wave cycle was 3 dB higher than that of the stationary noise. The noise was gated on 1 s prior to the beginning of each sentence, and was gated off 1 s after the end of each sentence. The average sentence duration was about 1.5 s.

#### Presentation

Three types of speech (voiced, whispered, and adjusted whispered), two types of noise (continuous and gated), and three SNRs (−6 dB, −3 dB, and 0 dB) were tested. The 320 sentences were grouped into 40 lists of 8 sentences and were tested in list-long blocks. Two blocks were tested per listener and condition. The presentation order of conditions and lists was randomized independently for each listener.

Subjects listened to sentences, presented diotically over Sennheiser HD650 headphones in a double-walled sound-attenuating booth, and typed the key words of each sentence into a text field within a graphical user interface. Each sentence had 3 key words, which were scored individually, so that an individual’s speech perception score in each condition and SNR was based on 48 possible words. Four non-musician listeners were tested only at −3 dB and 0 dB SNR in the whispered speech condition, so their data from that condition were not included in the analysis, thereby reducing the degrees of freedom in statistical models considering whispered speech data.

### Results

**Figure 1:**
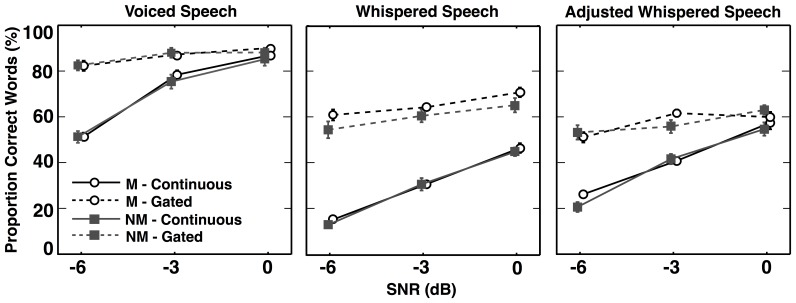
Mean proportion of correctly identified words as a function of signal-to-noise ratio (SNR). Musician (M; circles) and non-musician (NM; squares) data from continuous noise (solid lines) and gated noise (dashed lines) trials are included for each of the three speech types, shown in the three panels. No significant differences between the groups were observed. Error bars indicate +/−1 standard error (s.e.) of the mean.

The results with the voiced speech (VS) are shown in the left panel of Figure 1. These data show that the proportion of correctly identified words improved with increasing SNR, and that the improvement was more marked in the continuous noise than in the gated noise. Masking release, defined as the difference in scores between the continuous and gated noise, was greatest at the lowest SNR and decreased with increasing SNR, perhaps in part because the high performance at high SNRs led to some ceiling effects. The middle panel of Fig. 1 shows the proportion of words correctly reported in the whispered speech (WS) conditions (16 musicians; 13 non-musicians in this condition). All listeners reported fewer correct words in the WS condition, but more masking release was observed in this condition, especially at the highest SNRs. The right-hand panel shows results from the condition where the WS was adjusted to have the same spectro-temporal envelope as the VS. The pattern of masking release in this condition was similar to that found in the VS condition, but the generally lower proportion of correct words is more similar to that found in the WS condition. Here the decreased masking release at the high SNR cannot be attributed to ceiling effects. No systematic differences between the musician and non-musician groups were apparent in any of the conditions.

The proportion of correct words from all three speech conditions were transformed into rationalized arcsine units (RAU) [30], which were used as the dependent variable in a mixed-model analysis of variance (ANOVA), with speech type (VS, WS, and adjusted WS), SNR, and noise type (continuous and gated) as within-subject factors, and musical training as a between-subjects factor. Neither the main effect of musical training [F(1,27) = 0.96, p = 0.34], nor its interaction with SNR [F(2,54) = 0.05, p = 0.95], speech type [F(2,54) = 0.95, p = 0.39], or noise type [F(1,27) = 0.18, p = 0.68] was significant. Higher (3-way or 4-way) interactions with musical training also failed to reach significance (p>0.15 in all cases).

Other main effects were significant. As expected, there were main effects of SNR [F(2,54) = 283.1, p<0.001, η^2^ = 0.91], speech type [F(2,54) = 1012.3, p<0.001, η^2^ = 0.97], and noise type [F(1,27) = 496.4, p<0.001, η^2^ = 0.95], with interactions that were very similar to those found in an earlier study that investigated the differences between voiced and whispered speech in continuous and gated noise backgrounds [9]. Although IQ varied significantly between groups, it was not found to co-vary with data from any speech type or gating condition (averaged across SNRs; p>0.5 in all cases).

Finally, the data from the musicians were considered separately, and an analysis of covariance was undertaken, with within-subjects factors of speech type, SNR, and noise type, and a covariate of age of onset of musical training. No significant main effect of age of onset of musical training was found [F(1,14) = 0.015, p = 0.9], and no significant interactions with age of onset were identified [interaction with Speech Type F(2,28) = 0.51, p = 0.61; interaction with SNR F(2,28) = 2.9, p = 0.07; interaction with Gating F(1,14) = 2.1, p = 0.17; p>0.77 for all three-way and four-way effects]. The model was repeated with years of training as a covariate, which was also found not to be significant [F(1,14) = 0.063, p = 0.81], with no significant interactions with the other factors [interaction with Speech Type F(2,28) = 0.37, p = 0.70; interaction with SNR F(2,28) = 2.0, p = 0.16; interaction with Gating F(1,14) = 0.003, p = 0.96; p>0.1 for all three- and four-way interactions].

### Discussion

No significant effects of musical training were found. Although the lack of an effect of musical training with whispered speech is consistent with our initial hypothesis, the lack of an effect in normal (voiced) speech is not consistent with the expectation of a significant benefit of musical training in understanding speech in noise. In addition, contrary to our hypothesis, the gated noise did not result in a larger difference in performance between musicians and non-musicians than the continuous noise. Overall, no evidence was found in the current experiment to support the hypothesis of an association between musical training and improved speech perception in noise.

The aim of the adjusted WS condition was to eliminate periodic TFS information while maintaining the same spectro-temporal envelope information available in normal speech. A comparison of the results from the left and right panels suggests that this aim was successful: aside from an overall reduction in performance due to lack of voicing, the VS and adjusted WS conditions produced similar outcomes. In particular, at the highest SNR of 0 dB, the results from the continuous- and gated-noise conditions converged in both cases, supporting the hypothesis of Bernstein and Grant [23] that the amplitude distribution of normal speech helps account for why masking release is reduced at higher SNRs. The results also confirm the conclusion of Freyman et al. [9] that whispered speech continues to elicit masking release at higher SNRs (see middle panel of Fig. 1 with 0-dB SNR) because of its narrower distribution of amplitudes.

Our finding of no significant effect of musical training on understanding normal speech in noise seems at odds with an earlier report of Parbery-Clark et al. [14]. The criteria for distinguishing between musicians and non-musicians were relatively similar: in both studies, participants were normal-hearing native speakers of American-English with normal to above-normal intelligence. All musicians in both studies had 10 or more years of musical training (average of 14.9 years here, compared to 16 years in Parbery-Clark et al.). In this study, five of sixteen musicians began training aged 8–10 years, and the remaining 11 began at age 7 or younger, whereas all of the Parbery-Clark et al. musicians began at or before age 7. Based on this criterion, five of our musicians would not have qualified for the Parbery-Clark et al. study, despite all of our musicians being considered musicians by most definitions. The musicians in this study reported playing at least 5 hours a week, which is probably comparable to the Parbery-Clark et al. requirement of playing at least 3 times a week, although most of our subjects (14 of 16) reported playing 10 hours a week or more. Although the average number of years of musical training was slightly lower in our group, and the average age of onset was slightly higher, neither of these factors was found to be a significant covariate in our analysis, suggesting that these small differences are unlikely to explain the divergent results of the two studies.

The speech materials used in the two studies were quite different, so it remains possible that the advantage found by Parbery-Clark et al. was due to the specific clinical measures of speech understanding used in their study. Experiment 2 was conducted to further explore this apparent discrepancy by using the same standardized clinical speech materials and similar methods to those used by Parbery-Clark et al. [14]. In addition Experiment 2 provided a direct test of musical aptitude using a pitch-discrimination task.

## Experiment 2

### Rationale

The aim of this experiment was to determine whether standard clinical speech-in-noise tests would reveal advantages for our sample of musicians, despite the null results obtained with the methods and materials of Experiment 1. The tests were chosen to replicate as closely as possible the measures used by Parbery-Clark et al. [14], who reported small but significant differences between musicians and non-musicians. The two clinical speech-in-noise tests used were QuickSIN [31] and Adaptive HINT [32], which both have relatively high context and word predictability, whereas Experiment 1 used grammatically correct nonsense sentences with very low context and predictability. These clinical tests are also administered such that the subject and tester interact directly, possibly influencing performance via motivational differences between the groups.

Another possible reason why we may have found no differences between our samples of musicians and non-musicians relates to the perceptual skills of our musicians. To assess these skills, we measured F0 discrimination in both musicians and non-musicians. Several studies have shown that musicians typically have an advantage in F0 discrimination over untrained non-musicians [3]. Thus, in the absence of extended training in the lab, we would expect our non-musicians to exhibit higher (poorer) F0 difference limens than our musicians.

### Methods

Ten non-musicians and 13 musicians from Experiment 1 returned for Experiment 2 and were supplemented by 2 additional non-musicians and 1 additional musician for a total of 12 non-musicians and 14 musicians. The definitions of musicians and non-musicians were the same as for Experiment 1.

The QuickSIN test was administered at 70 dB SPL diotically over audiometric Telephonics 296D000-1 headphones to listeners in a double-walled sound-attenuating booth. Sentences spoken by a female voice embedded in 4-speaker babble were played in blocks of 6 sentences, beginning with an SNR of 25 dB and progressing to 0 dB in steps of 5 dB. Listeners repeated each sentence out loud to the tester via an intercom, and the tester marked how many of 5 key words were correct in each sentence. The correct words reported for each list of 6 sentences were summed and subtracted from 25.5. One practice block and eight test blocks were administered, and the results from the 8 test blocks were averaged to produce a final estimate of speech-in-noise SNR loss.

Syntactically and grammatically simple HINT sentences were presented diotically under headphones in an adaptive procedure. We tested only co-located speech and noise, as the spatially separated HINT-L and HINT-R conditions in Parbery-Clark et al. [14] produced a null effect of musical training. Sentences were organized into lists of 10 sentences each, and nine randomly selected lists were presented to each subject.

Speech-shaped noise preceded each sentence by 200 ms and continued for 100 ms after the conclusion of each sentence. The noise level was fixed at 65 dB SPL, and SNR was adaptively varied using a one-up-one-down procedure with step size of 1 dB and a starting SNR of 0 dB. Listeners repeated each sentence out loud to the tester, and the tester recorded responses as either correct (all words reported correctly; SNR adjusted down) or incorrect (any incorrectly reported words; SNR adjusted up). Threshold for each track was the mean speech level at the reversal points, and each listener’s threshold was calculated as the mean of the last 8 track thresholds (the first track was treated as practice).

Listeners also completed a pitch discrimination task. In each trial, they were sequentially presented with two complex tones, differing in F0, and were asked to identify which had the higher F0. Thresholds were estimated using an adaptive 2-down 1-up tracking procedure. Test signals were 300-ms harmonic complex tones, lowpass filtered at 600 Hz with a 12 dB/octave slope, gated on and off with 20-ms raised-cosine ramps, and presented at an overall level of 65 dB SPL. Thresholds were measured for nominal F0s of 110 and 210 Hz. In each trial, two complex tones were presented, with F0s geometrically centered on the nominal F0, and separated by an inter-stimulus interval of 500 ms. Feedback was provided after each trial. At the beginning of each adaptive track, the initial F0 difference (ΔF0) was 20% (expressed as a proportion of the lower of the two F0s). The value of ΔF0 was increased or decreased initially by a factor of 2. This factor was decreased to 1.26 after two reversals in the tracking procedure, and then was decreased again to a final step size of 1.12 after a further two reversals. Threshold was defined as the geometric mean value of ΔF0 at the last 6 reversals at this final step size. Each listener completed 6 tracks for each of the two baseline F0s, and thresholds for each listener were computed as the geometric mean across the 6 tracks.

### Results

**Figure 2:**
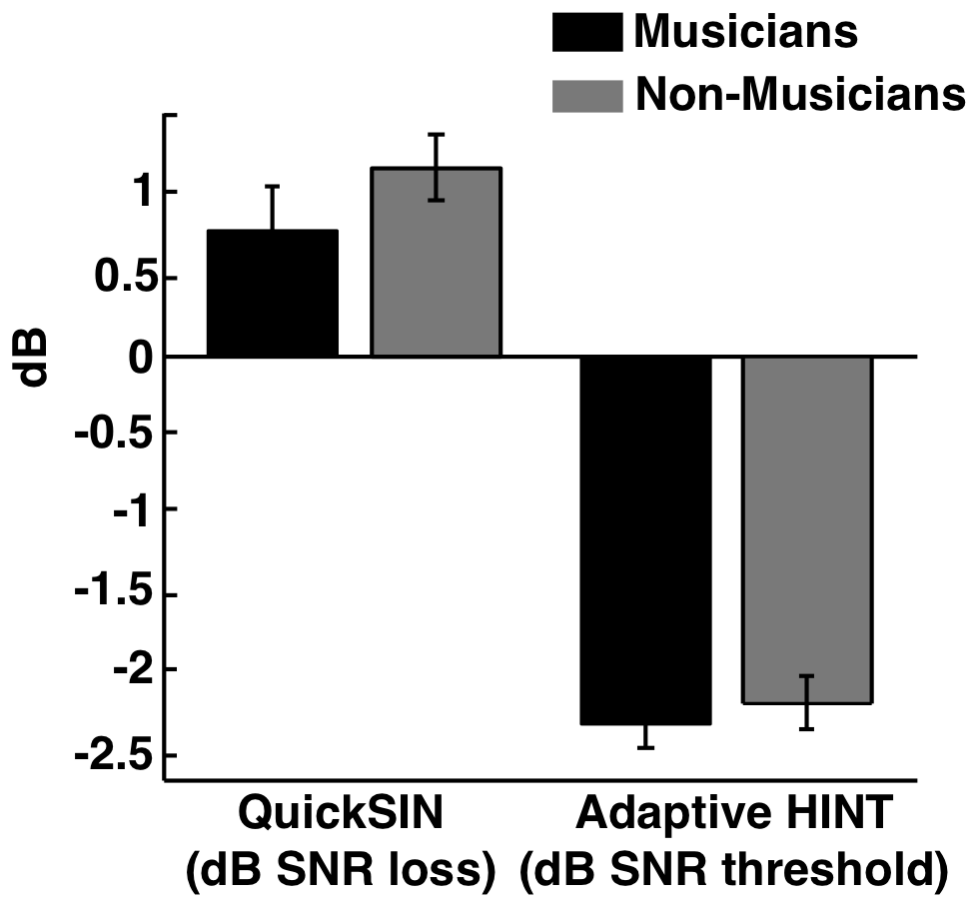
Average results for QuickSIN and Adaptive HINT measures of speech perception in noise. Black bars denote the average performance of musicians, and grey bars denote the average performance of the non-musician data. The QuickSIN measure (left group) indicates dB of SNR loss, relative to an ideal level of speech understanding in 4-speaker background babble. The Adaptive HINT measure (right group) indicates the threshold SNR for speech understanding in a continuous speech-shaped noise. In both cases, lower scores denote better performance. Error bars represent +/−1 s.e. of the mean.

Mean speech-in-noise SNR loss, measured with the QuickSIN test, is plotted for musicians and non-musicians in the left-most bars of Figure 2. An independent-samples t-test showed no significant difference in the mean SNR loss for musicians (mean = 0.80 dB), compared to the non-musicians (mean = 1.12 dB) [t(24) = 1.12, p = 0.28]. The right two bars in Figure 2 show the mean SNR thresholds for the musicians and non-musicians in the adaptive HINT test. The mean threshold for the musicians (mean = −2.3 dB) was not significantly different from the mean threshold for the non-musicians (mean = −2.2 dB) [t(24) = −0.56, p = 0.58].

**Figure 3:**
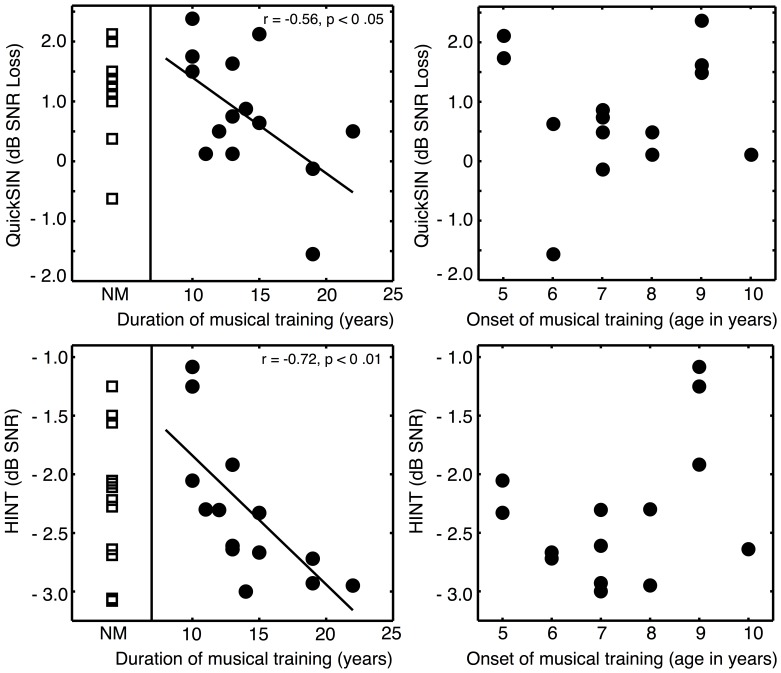
Scatter plots of individual musician speech-in-noise data as a function of years of musical training and age of onset of musical training. Individual non-musician data are shown in the far left column of each row. Distributions of musician and non-musician scores overlap, despite the significant within-group correlations with duration of musical training (but not age of onset).

Despite the lack of a group difference between musicians and non-musicians in QuickSIN and Adaptive HINT tests, a significant correlation within the musician group was found between years of training and both QuickSIN (R = −0.56, p = 0.037) and Adaptive HINT (R = −0.72, p = 0.0039), as was also reported by Parbery-Clark et al. [14]. These correlations are shown in Fig. 3, along with the data from the non-musicians. As can be seen in this graph, although a relationship is apparent within the musician group, the data from the non-musician group spans essentially the same range of performance. There was no significant relationship between the age of onset of training and either QuickSIN [R = 0.056, p = 0.85] or HINT [R = 0.35, p = 0.22]. The right-most panels of Fig. 3 illustrate the lack of a systematic relationship between speech-in-noise performance and age of onset of musical training. In contrast to the findings of Parbery-Clark et al., a significant correlation was observed between performance in the QuickSIN and HINT tests [R = 0.46, p = 0.018].

To increase statistical power, the results from the two tests were combined within a single ANOVA, with a within-subjects factor of test type (QuickSIN or HINT) and a between-subjects factor of musical training. There was a main effect of test type [F(1,24) = 403.18, p<0.001, η^2^ = 0.9], but neither the main effect of musical training [F(1,24) = 1.1, p = 0.31] nor its interaction with test type [F(1,24) = 0.67, p = 0.42] was significant. Thus, the additional statistical power achieved by pooling the data across the two tests did not result in a statistically significant effect of musical training.

**Figure 4:**
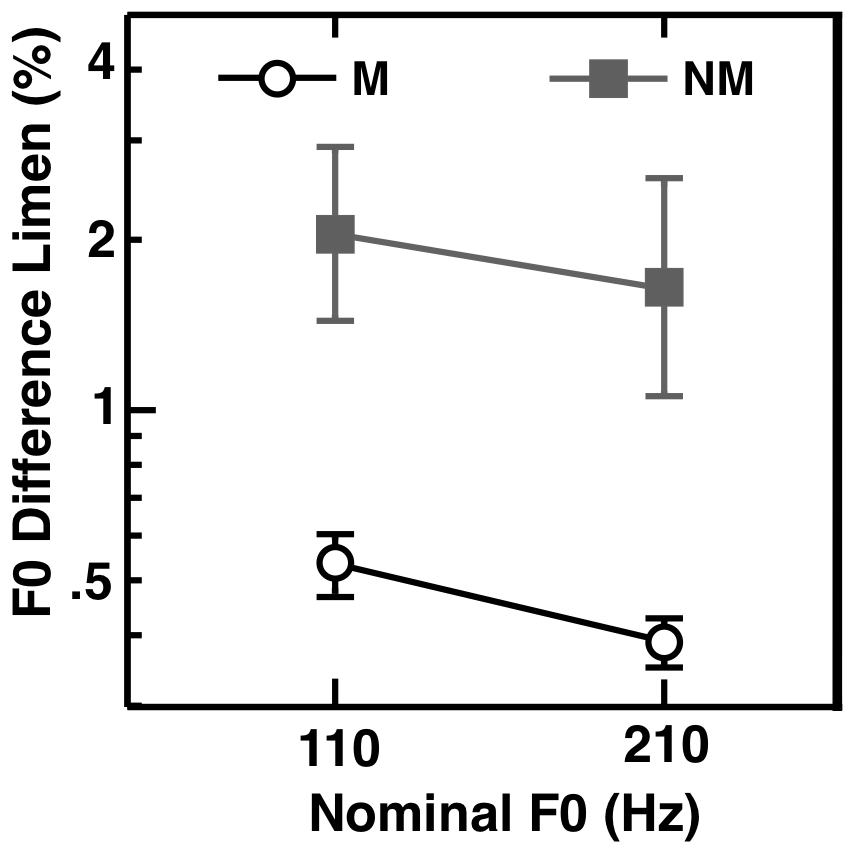
Fundamental-frequency difference limens. Musicians (open circles) demonstrate significantly better F0 discrimination limens than non-musicians (grey filled squares) for lowpass-filtered harmonic complexes with F0s centered around 110 Hz and 210 Hz. Error bars represent +/−1 s.e. of the mean.

Mean F0 discrimination thresholds for musicians and non-musicians are shown in Figure 4. A repeated-measures ANOVA confirmed that musicians had significantly lower (better) F0 difference limens than non-musicians [F(1,24) = 14.2, p = 0.001, η^2^ = 0.4]. The same ANOVA also showed that difference limens were somewhat lower for the 200-Hz F0 than for the 100-Hz F0 [F(1, 24) = 18.6, p<0.001, η^2^ = 0.44], and the lack of interaction between group and F0 [F(1,24) = 0.56, p = 0.46] suggests that this high-F0 advantage was similar in the two groups. There were no significant correlations between musicians’ F0 discrimination (averaged over the two measured frequencies) and the clinical speech in noise measures [QuickSIN: R = 0.013, p = 0.96; Adaptive HINT: R = 0.43, p = 0.12], the number of years of training [R = 0.45, p = 0.12], or the age of training onset [R = 0.076, p = 0.80].

Correlations between IQ and QuickSIN, HINT, age of onset, and years of training were all non-significant at the Bonferroni corrected level of 0.0125 [QuickSIN: R = −0.04, p = 0.87; HINT: R = −0.43, p = 0.04; age of onset: R = 0.29, p = 0.27; years of training: R = −0.31, p = 0.24].

### Discussion

Consistent with the results from Experiment 1, the clinical measures of speech understanding in noise did not reveal significant differences between musicians and non-musicians. The data show a small trend in the direction of the group differences seen in Parbery-Clark et al. [14], who found a small but statistically significant benefit of musical training in both the clinical speech tests used here, but in our case the differences were not statistically significant. A more detailed comparison of our data with those of Parbery-Clark et al. [14] revealed that the mean SNR loss in the QuickSIN test and adaptive HINT thresholds were both slightly higher for musicians in the present study than in Parbery-Clark et al. [14], whereas the thresholds for the non-musicians were very similar. We also note that the absolute differences between group scores in Parbery-Clark et al. [14] were relatively small (<1 dB) even in conditions that revealed a significant difference. It is also worth noting that only two of the four speech-in-noise tests conducted by Parbery-Clark et al resulted in significant group differences, even though the absolute differences in performance were similar. The two conditions that did not achieve statistical significance involved HINT sentences with the masker and speech spatially separated. Given the relative small effect size of the originally reported differences, it is perhaps not surprising that they are not robust to replication or small variations in conditions.

Although we strived to replicate as closely as possible the methods of Parbery-Clark et al., some small differences remained. For instance, with the QuickSIN test, Parbery-Clark et al. presented 4 lists per condition, whereas we presented 8 lists. The publishers of the QuickSIN test support presenting any number of lists between 1 and 9 and, of course, presenting more lists reduces measurement error. Aside from increased accuracy, there is no reason to suspect that our use of more lists influenced results: a repeated-measures ANOVA found no significant trend for improvement over the 8 lists [F(1,25) = 2.38, p = 0.14]. Another small difference was that Parbery-Clark et al. used insert earphones whereas we used supra-aural headphones. Both of these administration methods are supported by the test user guide, and there is no reason to suspect that different but calibrated headphones would result in systematic differences in performance.

Similar differences were present with the HINT test administration. We tested 9 lists per condition, whereas Parbery-Clark et al. presented only 3. Again, however, we found no significant effect of repetition number with the HINT sentences [repeated-measures ANOVA: F(1,25) = 1.77, p = 0.20], and gained narrower 95% confidence intervals for each subject through the use of more repeated measurements. The HINT sentences are often presented via headphones [32], [33], but can be presented over loudspeakers, particularly when the effects of spatial separation are being measured. Parbery-Clark et al. used a loudspeaker presentation but only found a statistically significant difference between musicians and non-musicians when the speech and noise were co-located, i.e., when there were no spatial differences between the stimuli. Thus, it seems unlikely that our use of headphones is responsible for the lack of significant group differences in this study.

The significant correlations between years of musical training and both HINT and QuickSIN scores contrast with the null group differences. In addition, the same correlations were not significant in Experiment 1. The main difference in materials between Experiments 1 and 2 was that the sentences in Experiment 2 contained relatively high semantic context. Thus, if at all, the benefit of musicianship may reflect higher-level cognitive advantages, rather than low-level perceptual processing benefits. However, even this speculation is called into question by the lack of a main group effect.

## General Discussion

Experiment 1 tested whether the putative advantage of musicians in understanding speech in noise could be explained in terms of superior coding of periodicity. The prediction was that the benefits of periodicity coding would not be observed with whispered speech, which lacks periodicity information. The results showed no significant differences between the musician and non-musician group in any of the conditions tested. Experiment 2 found a similar lack of a significant effect of musical training using clinical speech-in-noise tests that had been used in earlier studies [14], [15], although a benefit of musical training was observed in a pitch discrimination task.

Although our conclusions, based on the study of young adults, are inconsistent with those of two studies from another laboratory [14], [15] they seem to be in line with those of another study [34], which used a cross-sectional method to study the effects of age and musicianship on speech perception in noise. In that study, no consistent differences in speech understanding in noise between musicians and non-musicians below the age of 40 can be observed in their graph (their Fig. 4), although a trend for better performance in musicians appears in older listeners [34]. Our listeners (in both groups) were relatively young, so that it remains possible that a larger effect would have been observed had we studied older participants. On the other end of the developmental spectrum, a study carried out in children with and without musical training, also reported a difference in speech recognition in noise, as well as auditory working memory [35]. It remains unclear whether musical training enhances auditory working memory (or at least speeds its development), or whether children who have greater auditory working memory abilities are also more likely to display greater musical aptitude and interest, and so pursue music lessons.

One puzzling aspect of our data was the finding of an association between years of musical training and performance in the two clinical speech-in-noise tests, but no main effect of musical training. As with all studies relating musical training to other behavioral or neural measures, it is important to bear in mind the question of causality. Most discussions associating musicianship with improved performance in various tasks have implicitly or explicitly assumed some kind of causal relationship, for instance that musical training enhances neural coding or perceptual performance. As illustrated above in the case of children, an alternative hypothesis is that effort and performance in tasks, such as understanding speech in noise, covaries with cognitive and personality traits that also predict dedication to long-term musical training. This hypothesis is supported by evidence that length of musical training is correlated with cognitive variables and personality traits, and that personality variables account for at least as much variance as cognitive measures in predicting duration of musical training [36].

Our musicians obtained significantly better F0 discrimination thresholds than our non-musicians, consistent with earlier studies that compared untrained non-musicians to musicians [3], but we did not test factors such as auditory attention, auditory working memory, or temporal processing. As noted earlier, five musicians in Experiment 1 and six musicians in Experiment 2 began training later in life than would have been allowable in the Parbery-Clark et al. study. Also, although not an explicit criterion of the Parbery-Clark et al. study, all their musicians had first instruments of either piano or violin; we did not limit the instruments played by our sample, resulting in 11 instruments in our group. This heterogeneity may impact how intensely our musicians practiced when very young (wind instruments are more taxing for young children than violin or piano), but may be considered more representative of the class of individuals who are generally regarded as musicians within our society. In fact, some concern has been raised that attempts to generalize or find population differences may not be appropriate in a population as heterogeneous as individuals who can be classified as “musicians” [37]. Every musician participant in our group had dedicated significant time and resources to training for at least 10 years, and currently spends significant time and resources practicing music. If such individuals are not sufficiently well trained to exhibit a benefit of speech understanding in noise, then the effects may be of questionable generality. Overall, therefore, our results are not consistent with the idea that musical training, in its currently accepted definition, leads to auditory processing benefits that generalize to speech perception in noise. None of the differences between musicians and non-musicians were significant, even across our six different measures (voiced, whispered, continuous noise, gated noise, QuickSIN and HINT tests).

The lack of a robust effect of musical training on speech understanding in noise is in contrast to findings using tasks that have more direct commonalities with musical skills, such as mistuning detection, frequency discrimination [3], or analytic listening, as measured using informational masking [38]. However, it should be noted that with frequency or pitch discrimination, the large benefit observed in musicians can be eliminated with as little as 6–8 hours of training in non-musicians. Thus, at least for this very simple task, it takes relatively little time to become an “expert listener.” In comparison, most humans engage in listening to speech in noisy backgrounds on a daily basis, so that an argument could be made that we are all already highly experienced listeners in understanding speech in noise. This in turn offers a potential explanation for why musical training does not always generalize to speech understanding in noise.

The lack of an effect of musical training on speech perception in this study should, of course, not be interpreted to mean that there are no differences between musicians and non-musicians, especially in light of many reports of differences in personality, psychoacoustics, and neurophysiology. Instead, they illustrate that the differences may not always generalize beyond tasks that are closely related to music and that, if they do generalize to understanding speech in noise, the differences are at best small and not robust, at least in a heterogeneous, but representative, sample of young adult musicians.

## Conclusions

This study found no statistically significant differences between musicians and non-musicians in their ability to understand speech in noise under a number of conditions and using several measurement methods.

Musicians and non-musicians performed equivalently in low-context sentences using voiced speech, whispered speech, and whispered speech with the spectro-temporal envelope distribution matched to that of voiced speech. Both groups also performed equivalently in two clinical tests of speech understanding in noise using high-context sentences.

Both groups showed the same amounts of masking release when the steady-state noise was replaced with a square-wave gated noise. The similar masking release found for voiced and whispered speech supports the hypothesis that periodic temporal fine structure is not a significant factor in predicting speech masking release [9].

Our results do not preclude speech-in-noise differences between musicians and non-musicians over the lifespan [34], which may be related to personality and/or cognitive traits, rather than musical training *per se* [36]. However, the findings do suggest that the advantages of musical training may not functionally generalize to the domain of speech in noise perception, at least for young adults.

## Supporting Information

Audio File S1 **Example voiced speech sentence.**
(WAV)

Audio File S2 **Example whispered speech sentence.**
(WAV)

Audio File S3 **Example whispered speech sentence, adjusted to match the spectro-temporal envelope of the voiced speech sentence.**
(WAV)
